# A Singular Tumor

**Published:** 1870-11

**Authors:** John C. Storey


*A Singular Tumor.*—Mr. Editor:—As it has become quite fashionable to report every case which has any peculiarities to the writer himself, whether they appear so to others or not, I have thought to tell you of one which came under my observation a short time since, and should you deem it worthy, are welcome to give it publicity.

Mrs. B., aged 28 years, lymphatic temperament, general health good, sent for me some time during the summer (I did not at the time note the date) in haste, the message stating that she was bleeding profusely from the mouth. The distance being short, I was with her in a few moments, and found the hemorrhage coming from a polypoid tumor, or excrescence growing out from between the last two upper molar teeth of the left side. On the lingual surface the tumor was about the size of a small chinkapin, and was exceedingly vascular in structure, resembling very much in appearance a placenta.

The remedy in the case was to remove it, since there was no chance to arrest the hemorrhage without, and even if such were possible, it was best to remove it. There was no difficulty in detaching by tearing it away with the fingers. The bleeding ceased upon applying nitrate of silver, and all went well for the time. Now what renders the case interesting, none the less to medical men than to dentists, is the fact that the lady was about five months gone in pregnancy with her fifth child, and that the *polypus*, (for such it was,) lost no time in reproducing itself, which it continued to do, and be removed every four or six weeks, until Christmas morning, when she was delivered of a healthy child, at which time it stopped growing. I examined her mouth only a few days since and find nothing unnatural, save a slight bluish appearance about the gums, from which it had grown. Now, another fact worthy of notice is, that the above is the history also of the pregnancy next preceeding the one of which I write, and in this as in the last, it ceased to grow on her being confined, and commenced again on her becoming pregnant.

I would like to have the opinion of some one skilled in pathology, as to the rationale of this *affection*, since in summing it up I have arrived at the following very plain conclusion, that is, that pregnancy produces it, delivery cures it, and to prevent it, she must not again become pregnant. As to how and why such should be the case the writer ventures not an opinion.

John C. Storey, M. D., D. D. S.

